# Supplementary material for: Tuning crystal-phase of bimetallic single-nanoparticle for catalytic hydrogenation
Source: Nat Commun. 2022 Aug 5;13:4559. doi: 10.1038/s41467-022-32274-4 (PMC9355964; doi:10.1038/s41467-022-32274-4)
Supplement: Supplementary file 1 — Supplementary Information [file 41467_2022_32274_MOESM1_ESM.pdf]

Supplementary Information

Tuning crystal-phase of bimetallic single-nanoparticle for catalytic hydrogenation

Shuang Liu[1], Yong Li[1]*, Xiaojuan Yu[2], Shaobo Han[1], Yan Zhou[1], Yuqi Yang[3], Hao Zhang[3],

Zheng Jiang[3]*, Chuwei Zhu[4], Wei-Xue Li[4], Christof Wöll[2], Yuemin Wang[2]* & Wenjie Shen[1]*

[1]State Key Laboratory of Catalysis, Dalian Institute of Chemical Physics, Chinese Academy of Sciences, Dalian, China.

[2]Institute of Functional Interfaces, Karlsruhe Institute of Technology, Eggenstein-Leopoldshafen, Germany.

[3]Shanghai Synchrotron Radiation Facility, Shanghai Advanced Research Institute, Chinese Academy of Sciences, Shanghai, China.

[4]School of Chemistry and Materials Science, Hefei National Research Center for Physical Sciences at the Microscale, University of Science and Technology of China, Hefei, China.

*To whom correspondence should be addressed.

E-mail: yongli@dicp.ac.cn; jiangzheng@sinap.ac.cn; yuemin.wang@kit.edu; shen98@dicp.ac.cn

This file includes:

Supplementary Methods

Supplementary Figures 1-17

Supplementary References

Supplementary Methods

Crystal-phase mediation of PdCu nanoparticles

Monodisperse PdCu colloids were prepared by reducing palladium and copper cations in ethylene glycol and using oleylamine as the capping agent. Typically, 66.2 mg Na_2PdCl_4, 46.9 mg $CuCl_2·2H_2O$ and 1 ml oleylamine were dissolved into 100 ml ethylene glycol at room temperature under Ar. The mixture was heated to 393 K under stirring and maintained at that temperature for 20 min; further heated to 473 K and kept at that temperature for 2 h. The solid product was collected by centrifugation, washed with cyclohexane and acetone, and dispersed into cyclohexane (250 ml). Elemental analysis, using an inductively coupled plasma atomic emission spectroscopy (ICPS-8100 Spectro-meter), has identified the Pd/Cu molar ratio of 1/1. XRD and TEM analysis revealed that the PdCu colloids had an ordered body-centered cubic (B2) phase and a mean size of 8.0 nm (Supplementary Fig. 1).

Each PdCu colloid was then precisely coated with a silica shell using a water-cyclohexane reverse microemulsion method. PdCu colloids (37.4 mg), dispersed in cyclohexane (250 ml), were mixed with Triton X-100 (polyethylene glycol tert-octylphenyl ether, 80 ml) and ultra-sonically treated for 0.5 h at 293 K. Aqueous ammonia solution (29.4 wt.%, 5 ml) and aqueous hydrazine hydrate solution (80 wt.%, 5 ml) were added to the mixture, resulting in a pH value of 13. 9.35 g tetraethyl orthosilicate, mixed with 250 ml cyclohexane, was then added to the suspension, and stirred for 1 h. The solid product was collected by centrifugation, washed with ethanol, and dried at 323 K under vacuum for 12 h, yielding silica-coated PdCu colloid.

Tuning crystal-phase was done by treating the above silica-coated PdCu colloid with reactive gases (H_2/O_2) at 673-773 K. The chemically ordered body-centered cubic phase, donated as B2 particle, was prepared by treating the silica-coated PdCu colloid with H_2 at 673 K for 2 h. The face-centered cubic (fcc) phase, labeled as fcc particle, was obtained by calcining the B2 particle at 673 K in air for 4 h, followed by H_2 reduction at 773 K for 2 h. The fcc particle could also be prepared by directly calcining the silica-coated PdCu colloid at 673 K in air for 4 h and further reducing it with H_2 at 773 K for 2 h. N_2 adsorption–desorption isotherms revealed that the specific surface area was 177 m^2 g^{-1} for the B2 particle while 161 m^2 g^{-1} for the fcc particle,

contributed by the porous silica shells.

Structure characterizations

X-ray diffraction (XRD) patterns were recorded on a Rigaku D/MAX-2500/PC diffractometer using a Cu Kα radiation source that operated at 40 kV and 200 mA. In situ XRD measurements were done using a high-temperature chamber with the same instrument operated at 40 kV and 300 mA. 230 mg sample was pressed into a self-supporting wafer, mounted in the chamber, and heated from room temperature to 1073 K at a rate of 10 K min^{-1} under the flow of a 5.0 vol.% H_2/N_2 mixture (50 ml min^{-1}).

Transmission electron microscopy (TEM) images were taken over a Hitachi 7700 microscope operated at 100 kV; high-resolution TEM (HRTEM) images were recorded on a FEI Tecnai G2 F30 S-Twin microscope operated at 300 kV. Aberration-corrected high-angle annular dark field scanning transmission electron microscopy (HAADF-STEM) images were acquired over a JEOL-ARM 300F microscope operated at 300 kV using a low-dose rate and adopting the collector angle of 68-280 mrad with a probe convergence semi-angle of 24 mrad. To avoid the damage by electron beam irradiation, the sample was exposed to the electron beam only during the image acquisition. Energy dispersive X-ray spectroscopy (EDS) elemental mappings over the individual particles were collected using a JED-2300 T spectrometer. The specimen was prepared by ultrasonically dispersing the powder sample in ethanol, depositing droplets of the suspension onto lacey carbon-coated gold grids, and drying in air.

Environmental TEM (E-TEM) observation on the PdCu particles under H_2 and/or H_2/C_2H_2 was done using an aberration-corrected Titan Themis ETEM G3 operated at 300 kV with an electron dose rate of 1000 e $Å^{-2}$ s^{-1}. The sample was dispersed into ethanol, and the suspension was deposited onto a thermal E-chip that is equipped with a thin silicon nitride membrane. The sample was pretreated with H_2 at 673 K for 30 min. The images were acquired at 303 K and under 1 mbar H_2 or H_2/C_2H_2 (molar ratio of 1/1) using a one-view camera with 4k × 4k pixels.

Image simulation was done using the JEMS software package and the Multi-slice module; the microscopy parameters used for the simulations were the same as those for E-TEM imagining.

X-ray absorption structure (XAS) spectra of Cu and Pd K-edges were measured at the BL14W1 beamline at Shanghai Synchrotron Radiation Facility, China. The X-ray was mono-chromatized by a double-crystal Si (311) monochromator. The storage ring was operated at 3.5 GeV with a current of 230 mA. The sample (100-250 mg) was pressed into a self-supported wafer and mounted into a reaction cell, where it was treated with a 5.0 vol.% H_2/N_2 mixture (50 ml min^{-1}) at 673 K for 1 h, and the spectra were then recorded at 343 K. EXAFS data were processed according to the standard procedure using Athena and Artemis modules of the IFEFFIT software packages[1]. Contributions from different coordination shells were discriminated using a Hanning windows (dk = 1.0 $Å^{-1}$). Quantitative curve-fittings were done in the R-space (Cu 1.0-3.1 Å, Pd 1.0-3.3 Å) within a Fourier transform k-space range (3.0-11.4 $Å^{-1}$ for Cu and 3.0-13.5 $Å^{-1}$ for Pd). The overall amplitude reduction factor, S_0^2, was set at 0.77 for Pd K-edge and 0.92 for Cu K-edge. Simulations on the XANES spectra of Cu K-edges were performed with the FDMNES code in the framework of real-space full multiple-scattering (FMS) scheme using the Muffin-tin approximation for the potential[2].

IR experiments were done with a dedicated and sophisticated ultrahigh vacuum Fourier transform IR spectroscopy (UHV-FTIRS) using CO as the probe molecule, which has been demonstrated to be a highly surface-sensitive and non-destructive approach[3-5]. The sample (200 mg) was pressed into an inert metal mesh and mounted on a sample holder that was specially designed for the transmission model IR measurement. Exposure to CO was done by using a leak-valve-based directional doser connected to a tube (2 mm, inner diameter) that terminated 3 cm from the sample surface. The sample was treated with H_2 at 673 K for 1 h, exposed to CO at 110 K and gradually heated to 460 K at a rate of 3 K min^{-1}. IR data were accumulated by recording 1024 scans with a resolution of 4 cm^{-1}. Peak fittings on the spectra

of CO adsorbed on Pd and Cu sites were performed by the Gaussian function. Before each exposure, a spectrum of the clean sample was recorded as the background reference.

Microcalorimetric measurements of H_2 and C_2H_2 adsorption on the sample were done at 313 K with a BT 2.15 heat-flux calorimeter. The sample was treated with a 5.0 vol.% H_2/N_2 mixture at 673 K for 1 h, and then evacuated for 0.5 h at the same temperature to remove the physically adsorbed species. After being cooled down to room temperature, the sample was refilled with He, sealed in a thin quartz tube, and transferred into the calorimetric cell that was immersed within the isothermal calorimetric block.

Theoretical calculations

Density functional theory (DFT) calculations were done using the Vienna *ab initio* Simulation Package (VASP) code[6]. The electron correlation was performed within the generalized gradient approximation (GGA)[7] using a Perdew-Burke-Ernzerhof (PBE) exchange-correlation functional[8]. The kinetic energy cutoff was 400 eV for the plane-wave expansion. The geometry convergence tolerance for energy change and max force were 10^{-5} eV and 0.01 eV $Å^{-1}$, respectively. Zero-point vibrational energy corrections were included and calculated as half the sum of the energies of all vibrational modes:

$$E_{ZPE} = \frac{1}{2} R \sum_K \frac{h v_K}{k_B} \quad (1)$$

where R is the ideal gas constant, h is the Planck constant, v_K is the vibrational frequency for the mode K, k_B is the Boltzmann constant.

Crystal orbital Hamiltonian population (COHP) analysis was performed with the Local-Orbital Basis Suite Towards Electronic-Structure Reconstruction (LOBSTER) software (2.1.1 package)[9-11]. The orbital-resolved wave functions were reconstructed *via* projection of the delocalized PAW to localized atomic-like basis sets. Basis sets given by Koga[12,13], with additional functions fitted to atomic VASP PBE wave functions, were used[14].

The transition states of H_2 dissociation were located using the dimer method[15] and the convergence criteria were set to 0.05 eV Å^{-1}. The obtained saddle points were further confirmed by a single imaginary frequency along the reaction direction. The lattice constants of B2 and fcc phases were calculated to be 3.016 and 3.806 Å, respectively. An ordered $L1_0$-type (the AuCu-type) was used to simulate the disordered fcc (111) facet, based on the same space packing pattern of metal atoms[16,17]. To model the B2 and fcc PdCu surfaces, a 4-layer (3×2) B2(110) and a 4-layer (1×2) fcc(111) slabs were utilized and a 15 Å vacuum was introduced along z-axis to avoid artificial interactions. The bottom 2 layers were fixed in their bulk position while other metal atoms and adsorbates allowed to relax. The Brillouin zone was sampled by a Monkhorst-Pack (4×4×1) and (7×7×1) k-point mesh[18] for the B2(110) and fcc(111) slabs, respectively. The adsorption energies were calculated as:

$$E_{\mathrm{ads}}(\mathrm{molec}) = E_{\mathrm{molec/slab}} - E_{\mathrm{slab}} - E_{\mathrm{molec(g)}} \quad (2)$$

where $E_{\mathrm{molec/slab}}$, E_{slab} and $E_{\mathrm{molec(g)}}$ are the total energies of adsorbed system, clean slab, and adsorbate in the gas phase, respectively. The adsorption energy of atomic H is with respect to $1/2H_2(g)$.

Catalytic tests

Selective hydrogenation of acetylene was performed with a continuous-flow fixed-bed quartz tubular reactor (inner diameter, 6 mm) at atmospheric pressure. 50 mg catalyst (40–60 mesh) was pretreated with a 5.0 vol.% H_2/N_2 mixture (50 ml min^{-1}) at 673 K for 1 h. After being cooled down to room temperature (298 K), the catalyst was exposed to the reaction gas (1.0 vol.% C_2H_2/0.5–2.0 vol.% H_2/He, 50 ml min^{-1}) that was introduced *via* a mass flow controller. The outlet from the reactor was analyzed online using a gas chromatograph equipped with a thermal conductivity detector and a flame ionization detector. C_2H_4 was the main product while C_2H_6, C_4H_6 and C_4H_8 were the minor byproducts. The selectivity towards ethylene was calculated based on carbon number. The conversion rate of acetylene was measured by

controlling the conversion of acetylene below 20% *via* varying the flow rate of the reaction gas or the weight of the catalyst. The apparent activation energy was obtained in the temperature range 268-318 K at H_2/C_2H_2 ratio of 1/1-2/1. The reaction orders with respect to hydrogen and acetylene were determined at 298 K by adjusting the concentrations of H_2 (0.5–3.0 vol.%) and C_2H_2 (1.0–3.0 vol.%) in the feed gases.

Supplementary Figures

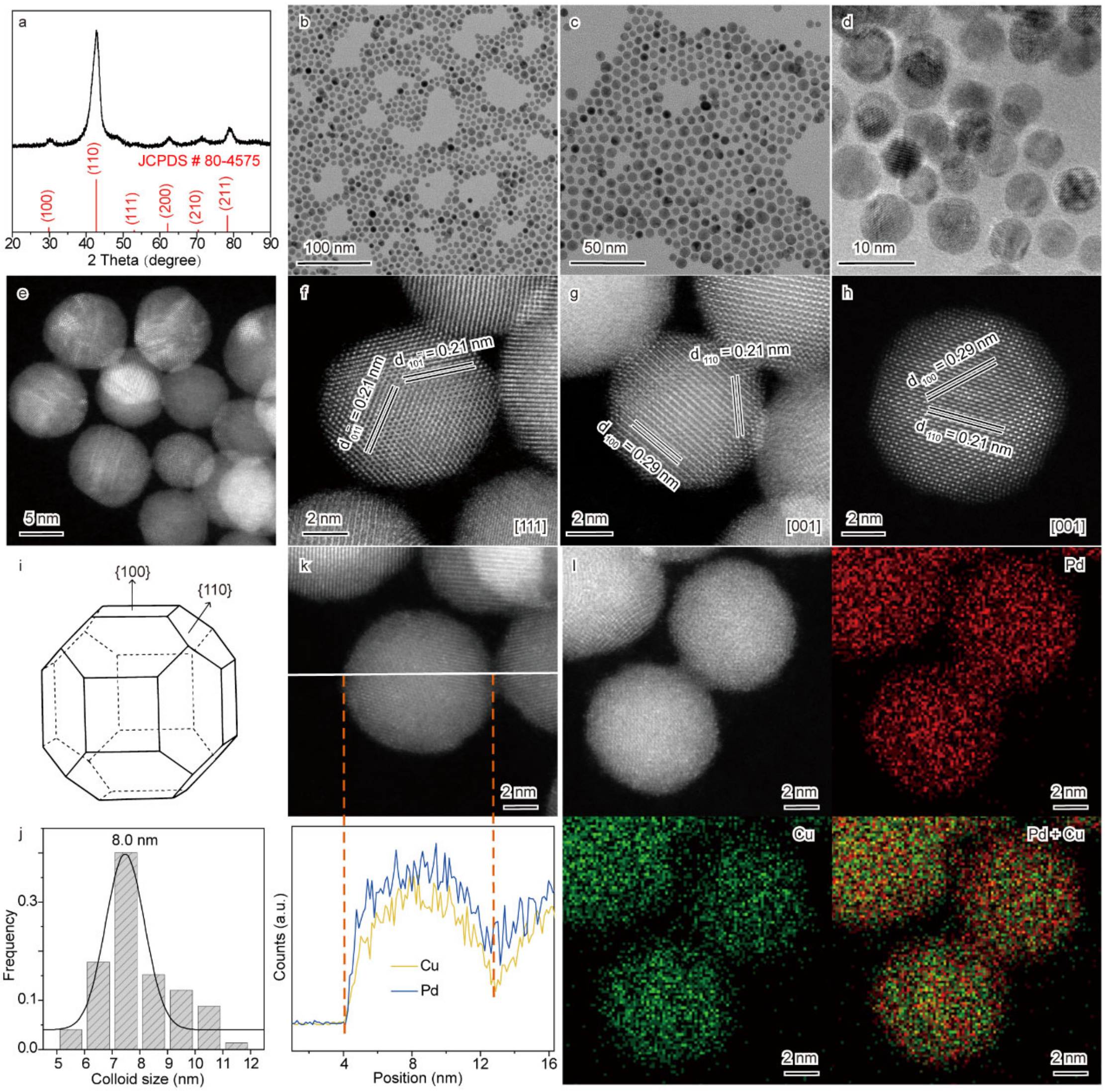

Supplementary Figure 1. Structure of the as-synthesized PdCu colloids. a, XRD pattern, showing the diffraction lines of the chemically ordered body-centered cubic structure. **b-h**, TEM/STEM images taken along different orientations: as viewed along the [111] direction (**f**), the two lattice spacings of 0.21 nm with a dihedral angle of 120° indicate the {110} facets; the lattice spacings of 0.29 and 0.21 nm with a dihedral angle of 45°, viewed along the [001] direction (**g, h**), index the {100} and {110} facets, respectively. **i**, Morphology illustration, depicting that the colloid is enclosed by twelve {110} facets and six {100} facets; the {110} facets is constructed by alternatively arranged Pd and Cu atoms while the {100} facets is terminated by either Pd or Cu atoms. **j**, Size distribution that is determined by counting 1005 colloids, giving the mean size of 8.0 nm within a very tight range (5-12 nm). **k**, Energy dispersive X-ray line scanning of a single colloid, evidencing the uniform distribution of Pd and Cu atoms from the outer surface to the bulk center. **l**, Elemental mapping crossing individual colloids.

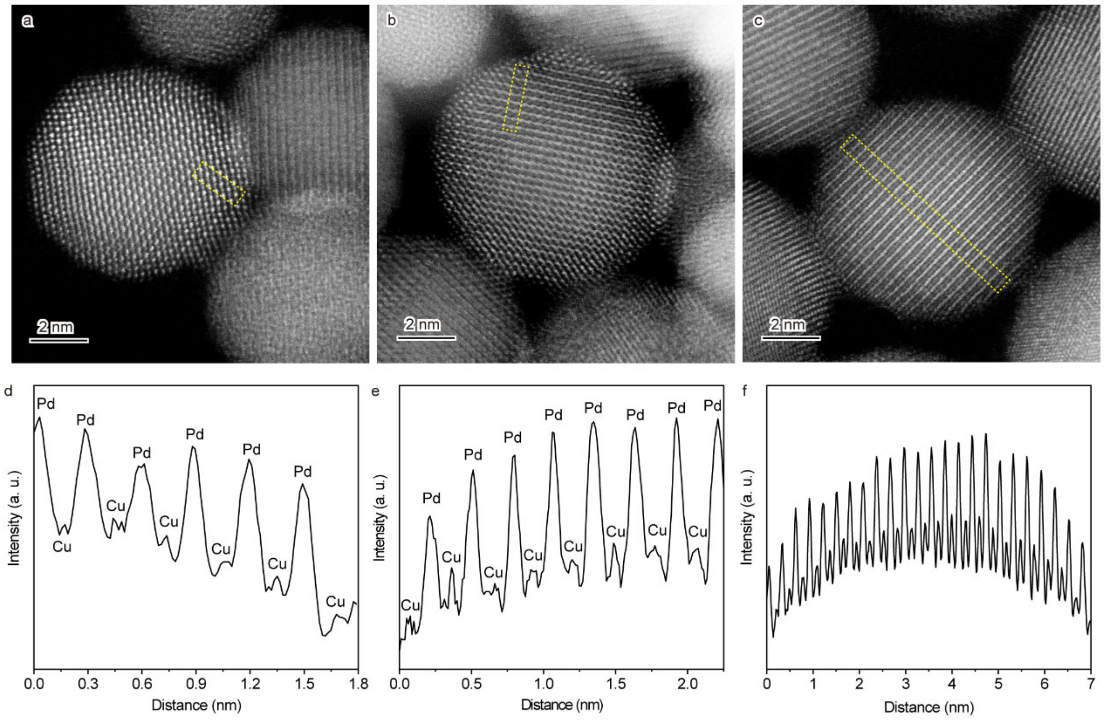

Supplementary Figure 2. Atomic arrangement of the as-synthesized PdCu colloids. a-c, STEM images, showing the orderly arranged Pd and Cu atoms. **d-f**, Intensity profiles taken along the rectangular regions in **a-c**, respectively, illustrating that Pd and Cu atomic columns are populated alternatively.

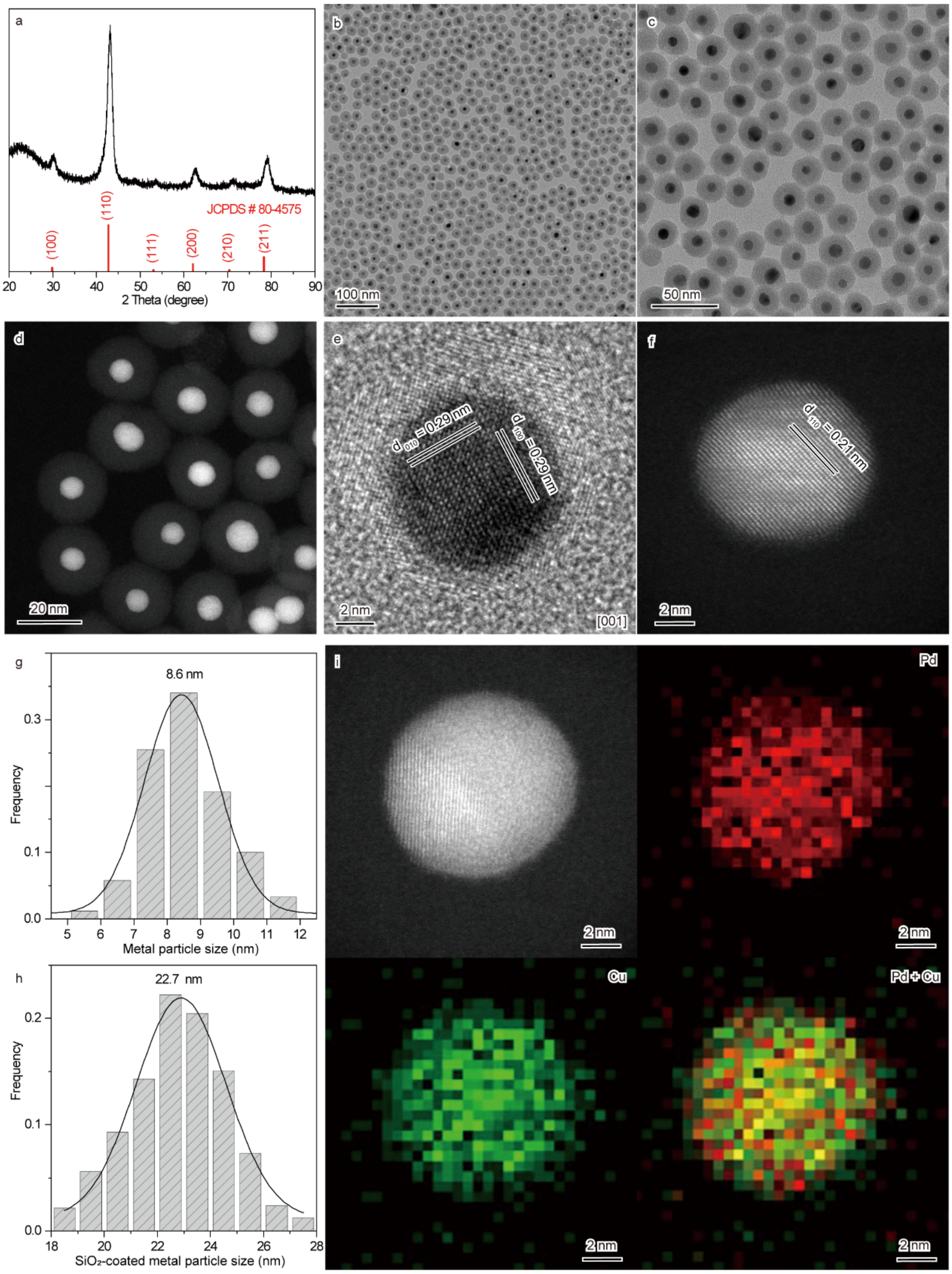

Supplementary Figure 3. Structure of the B2 particle. a, XRD pattern, displaying the diffraction lines of the chemically ordered body-centered cubic structure. **b-f**, TEM/STEM images, evidencing that each metal particle is coated by a silica shell and the {110} facets, indexed by the lattice spacing of 0.21 nm, are dominantly exposed. **g, h**, Size distributions derived by counting around 1300 particles, showing that the metal core has a mean size of 8.6 nm while the silica shell is 7.1 nm thick. **i**, Elemental mapping crossing a single particle, illustrating the uniform distribution of Pd and Cu atoms.

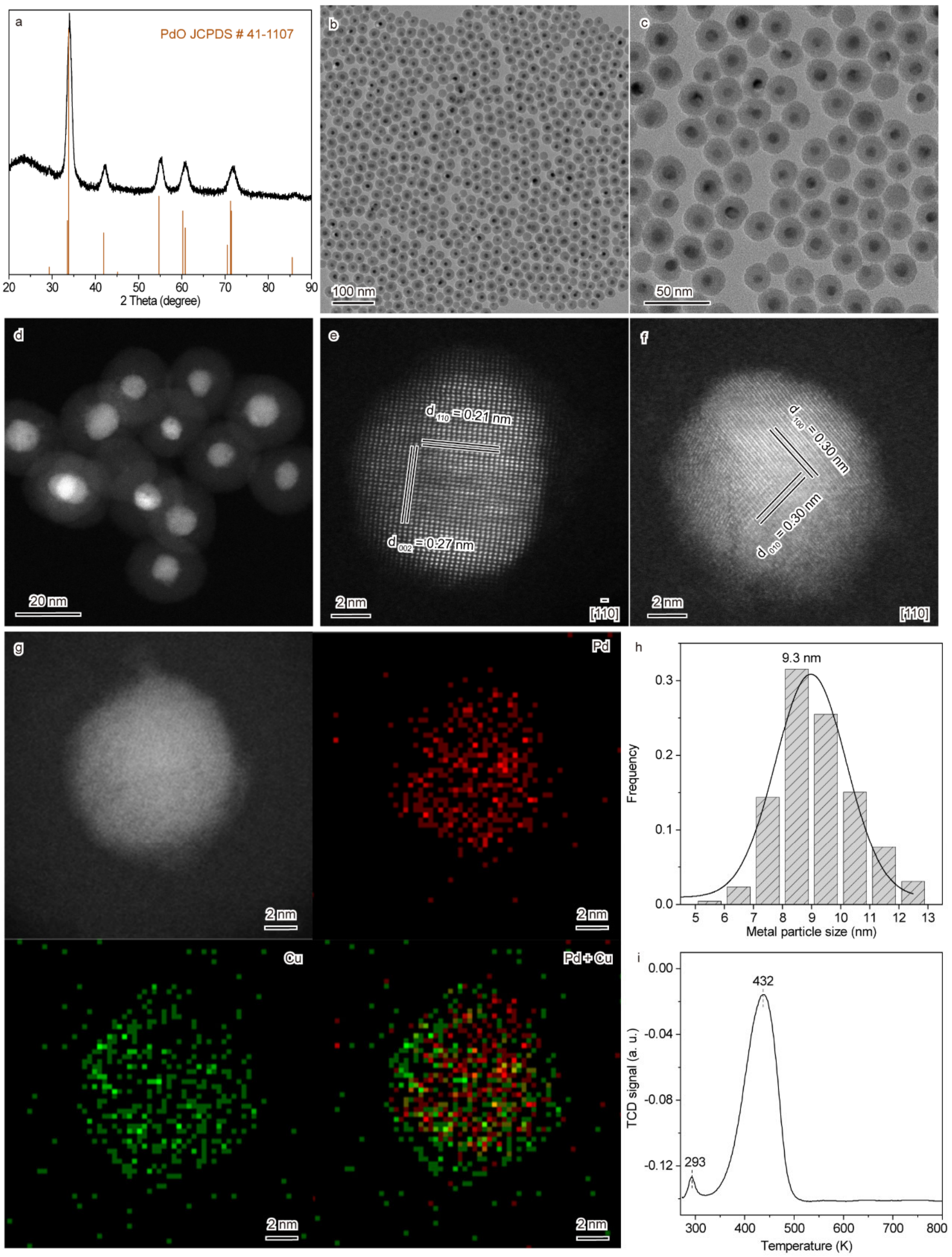

Supplementary Figure 4. Structure of the intermediate sample obtained by oxidizing the B2 particle at 673 K. a, XRD pattern, displaying the diffraction lines of PdO. **b-f**, TEM images, showing crystallized PdO and amorphous CuO or $PdCuO_x$. **g,** Elemental mapping crossing a single particle, illustrating the distributions of Pd and Cu atoms. **h**, Size distribution derived by counting around 800 particles, showing that the oxidized metal core has a mean size of 9.3 nm. **i**, H_2-TPR profile, illustrating that the oxidized particles are fully reduced into metallic states at temperatures below 500 K.

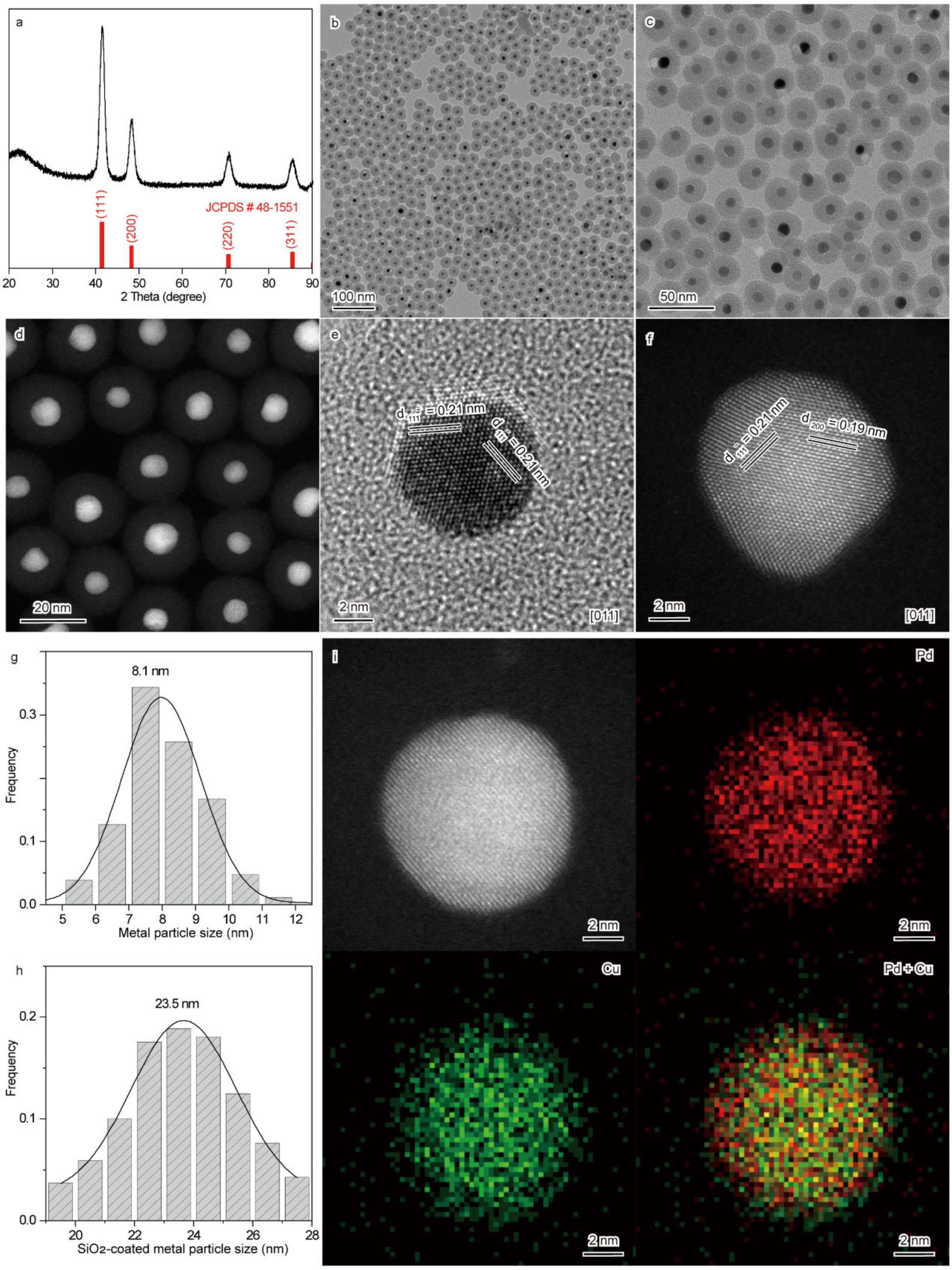

Supplementary Figure 5. Structure of the fcc particle. a, XRD pattern, showing the diffraction peaks of the face-centered cubic phase. **b-f**, TEM/STEM images, indicating the uniform coating of each metal particle by a silica shell and the dominantly exposed {111} facets as featured by the two lattice spacings of 0.21 nm with a dihedral angle of 110° and the minor {100} facets indexed by the lattice spacing of 0.19 nm. **g, h**, Size distributions determined by counting about 1100 particles, suggesting that the particle consists of a metal core of 8.1 nm and a silica shell of 7.7 nm. **i**, Elemental mapping crossing a single particle, showing the random mixing of Pd and Cu atoms.

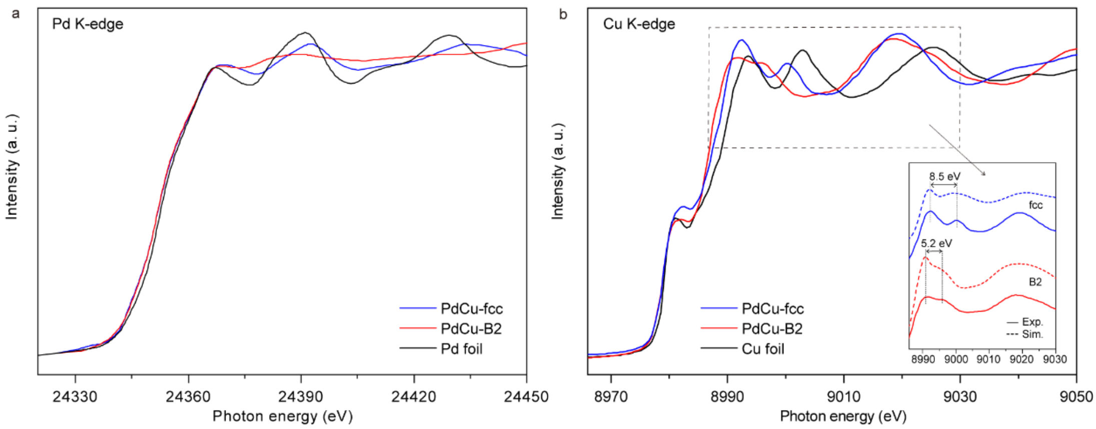

c

Atom	Bond	CN	R (Å)	σ^2 ($\times 10^{-3}$Å^2)	ΔE_0 (eV)	R factor (%)
Cu	Cu-Pd	5.9 ± 0.1	2.57 ± 0.01	8.6 ± 0.3	0.9 ± 0.5	0.4
	Cu-Cu	6.0 ± 2.1	2.92 ± 0.03	17.7 ± 4.0	-10.1 ± 0.6	
Pd	Pd-Cu	6.2 ± 0.3	2.56 ± 0.01	6.1 ± 0.1	-0.1 ± 0.6	0.2
	Pd-Pd	5.5 ± 0.4	2.98 ± 0.01	19.1 ± 2.0	6.8 ± 0.8	

d

Atom	Bond	CN	R (Å)	σ^2 ($\times 10^{-3}$Å^2)	ΔE_0 (eV)	R factor (%)
Cu	Cu-Pd	4.3 ± 1.5	2.61 ± 0.01	9.3 ± 0.1	2.9 ± 0.4	0.5
	Cu-Cu	5.7 ± 1.5	2.60 ± 0.01	16.4 ± 0.5	-0.2 ± 1.3	
Pd	Pd-Cu	4.7 ± 0.8	2.60 ± 0.03	4.3 ± 1.4	-1.4 ± 3.6	0.7
	Pd-Pd	5.3 ± 0.8	2.66 ± 0.02	8.0 ± 3.9	-1.5 ± 1.5	

CN, coordination number; R, the internal atomic distance; σ^2, Debye-Waller factor; ΔE_0, the edge-energy shift.

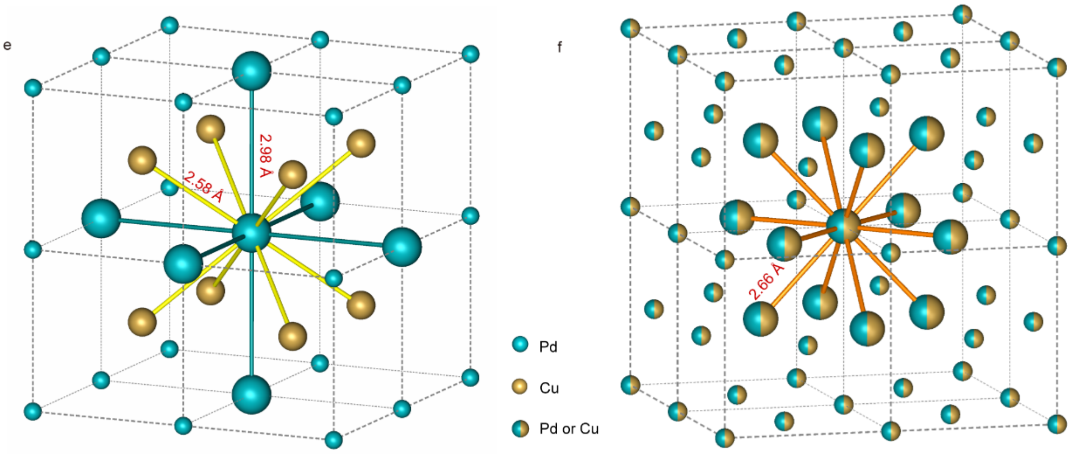

Supplementary Figure 6. XAFS analysis on the PdCu particles. a, **b**, Normalized XANES spectra of Pd (**a**) and Cu (**b**) K-edges; the inset in **b** illustrates the experimental data and the simulated results. **c**, **d**, EXAFS fitting parameters of Cu and Pd K-edges in the B2 (**c**) and fcc (**d**) particles. **e**, **f**, Bonding distances in the lattice cells of the B2 (**e**) and fcc (**f**) phases. The sphere size is intentionally lessened to highlight the coordination environments of Pd and Cu atoms. The bronze and cyan spheres in **e** denote Cu and Pd atoms, respectively, while the sphere with a mixed color in **f** means the random siting of Pd or Cu atoms.

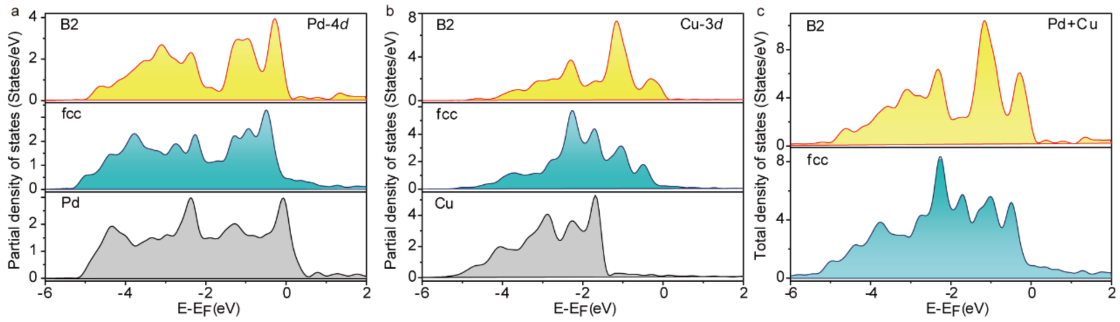

Supplementary Figure 7. Density of states of PdCu phases. a, **b**, Partial contributions of Pd-4*d* (**a**) and Cu-3*d* (**b**) to the total DOS for bulk B2 and fcc phases. The corresponding density of states of Pd and Cu bulks (grey) are shown as references. The resonance *d*-orbitals under the Fermi level evidence the electronic hybridization of Pd and Cu. **c**, The total density of states of Pd and Cu in B2 and fcc phases. The Pd *d*-band center in B2 phase is -2.08 eV in contrast to -2.34 eV in fcc phase. The Cu *d*-band in B2 phase also has a higher energy than that in fcc phase (-1.79 *vs* -2.07 eV). The upshifts of *d*-band centers of both Pd and Cu in the ordered B2 phase towards the Fermi levels demonstrate the chemically active nature, leading to a stronger interaction with adsorbates.

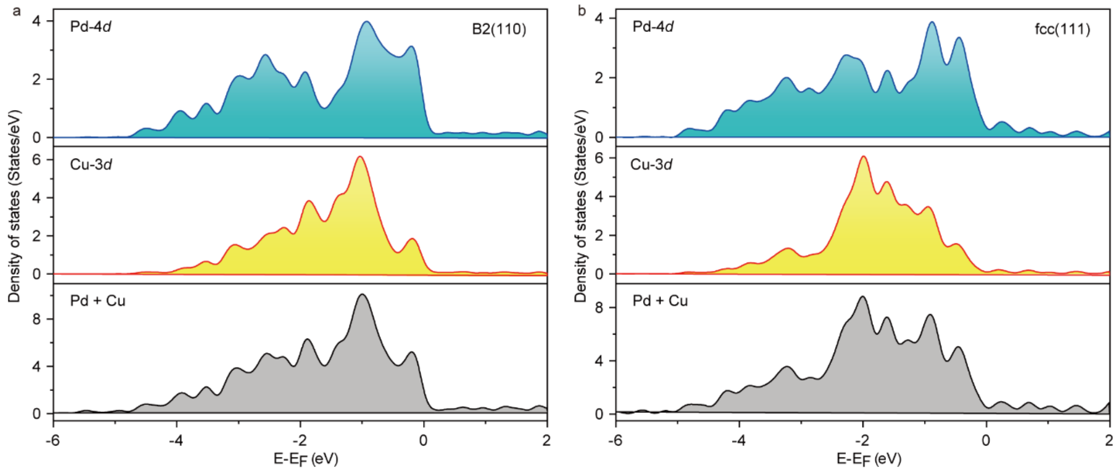

Supplementary Figure 8. Density of states of PdCu facets. a, **b**, Partial contributions of Pd-4*d* and Cu-3*d* to the total DOS of B2(110) **(a)** and fcc(111) (**b**) facets. The five hybridization peaks at -3.53, -3.06, -1.86, -1.03 and -0.20 eV show the strong Pd-Cu bonding by orbital hybridization over B2(110). The overall DOS of fcc(111) consists of hybridizations of Pd-4*d* and Cu-3*d* at -3.24, -1.60, -0.95 and -0.49 eV. Pd and Cu atoms on B2(110) have valence *d*-band centers at -1.74 and -1.59 eV, respectively; but the corresponding valence *d*-band centers over the fcc (111) surface locate at -1.96 and -1.84 eV.

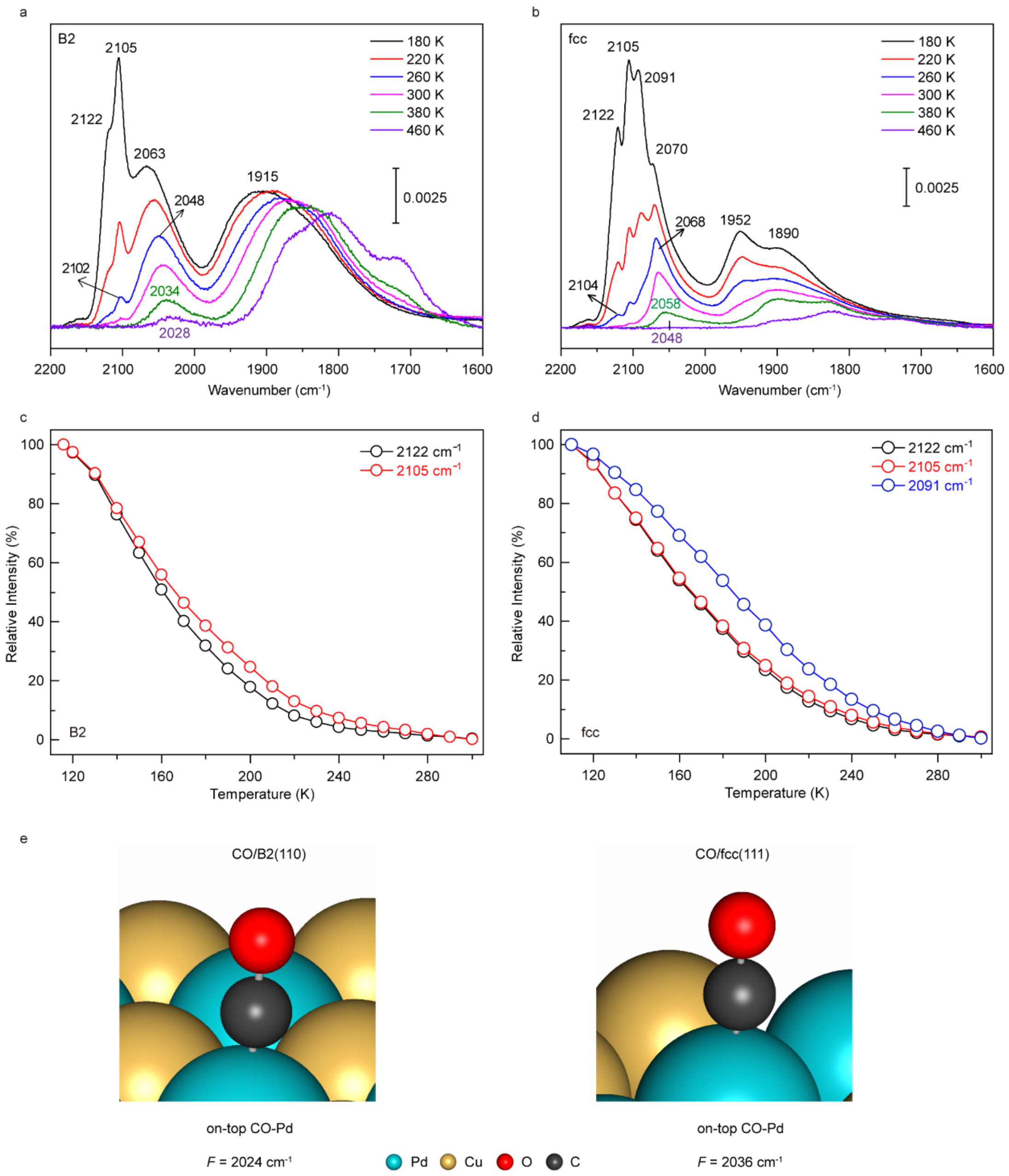

Supplementary Figure 9. Temperature-programmed IR spectra of CO adsorption on the PdCu particles. **a**, **b**, IR spectra recorded by exposing the samples to CO at 110 K and then gradually heated to 460 K at a rate of 3 K min^{-1}. **c**, **d**, Intensity evolution of Cu-related IR bands on the B2 (**c**) and fcc (**d**) particles. **e**, Calculated frequencies for the on-top CO-Pd bands on B2(110) and fcc(111) surfaces.

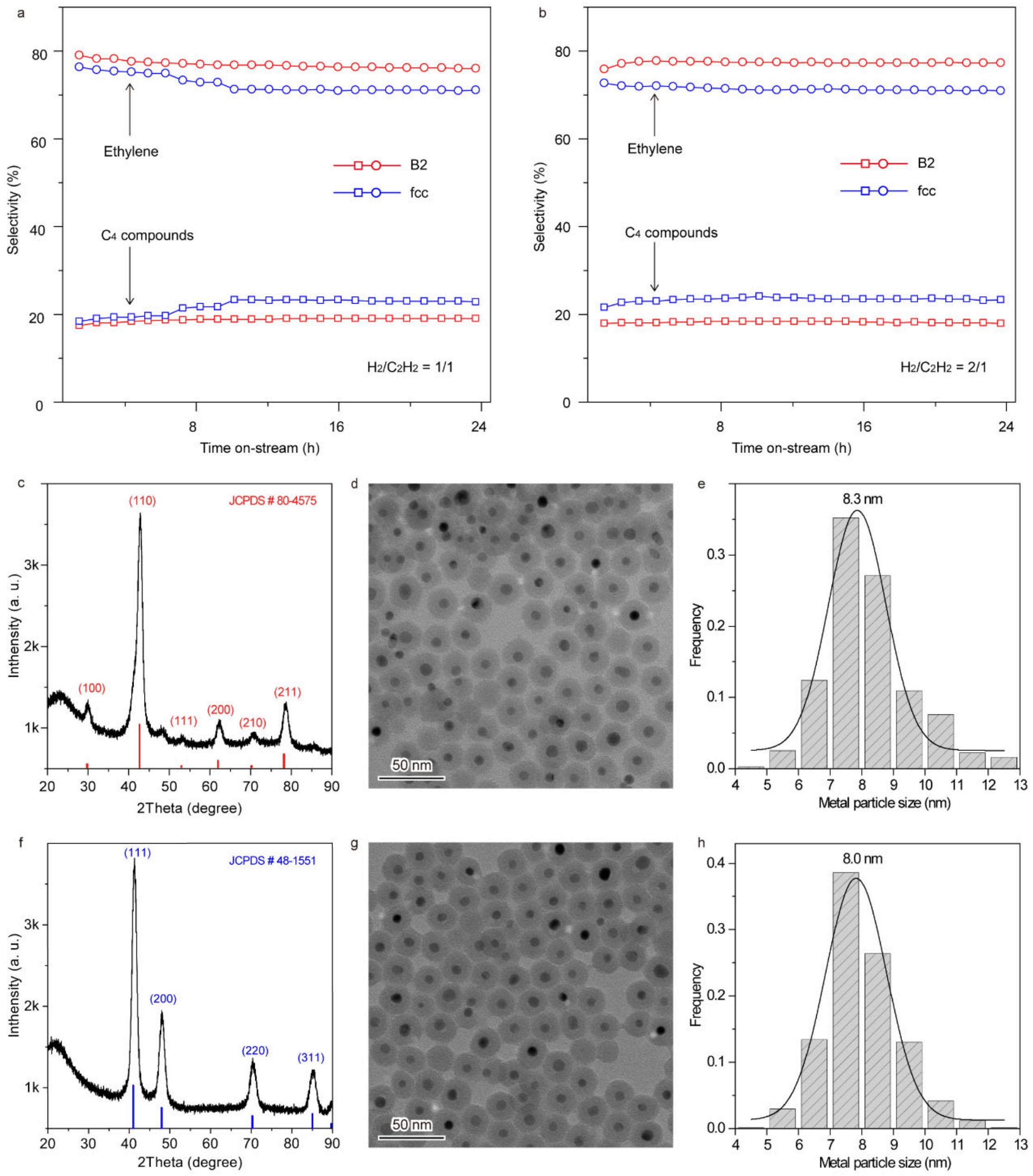

Supplementary Figure 10. Acetylene hydrogenation on the PdCu particles. a, **b**, Selectivities of the products at H_2/C_2H_2 molar ratios of 1/1 (**a**) and 2/1 (**b**). C_2H_4 is the main product (70–80%) while C_4 compounds (18–23%) and C_2H_6 (4–5%) are the by-products. **c-e**, XRD pattern, TEM image and size distribution of the spent B2 catalyst; **f-h**, XRD pattern, TEM image and size distribution of the spent fcc catalyst. Size distributions are derived by counting around 800 particles. The catalysts are tested for acetylene hydrogenation at 298 K for 24 h with a feed gas of 2%H_2/1%C_2H_2/He. XRD pattern of the spent B2 sample showed minor reflections that might be related to fcc phase.

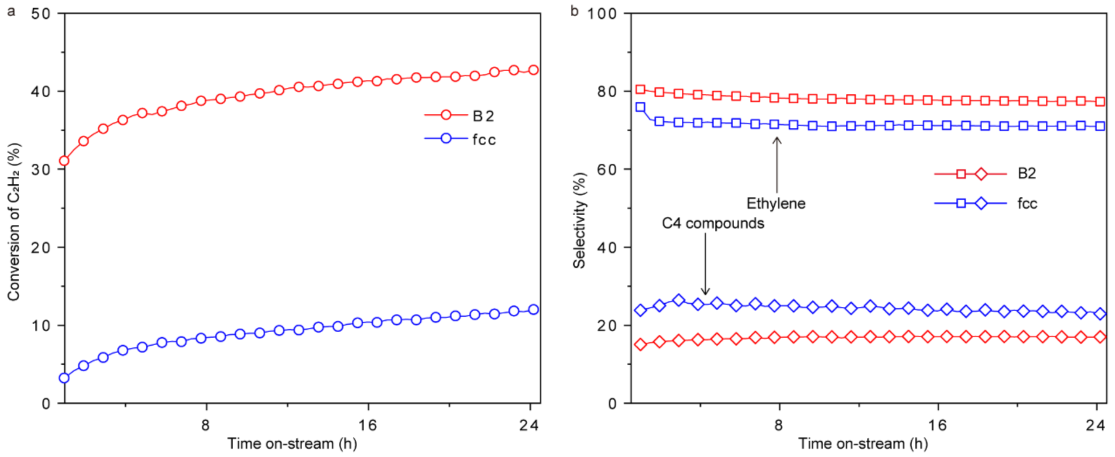

Supplementary Figure 11. Acetylene hydrogenation on the PdCu particles. a, Conversion of acetylene; b, Selectivities of the products. The reactions are performed at 298 K with a feed gas of 2%H_2/1%C_2H_2 /1%C_2H_4/He. C_2H_4 is the main product (70–80%) while C_4 compounds (17–25%) and C_2H_6 (4–5%) are the by-products.

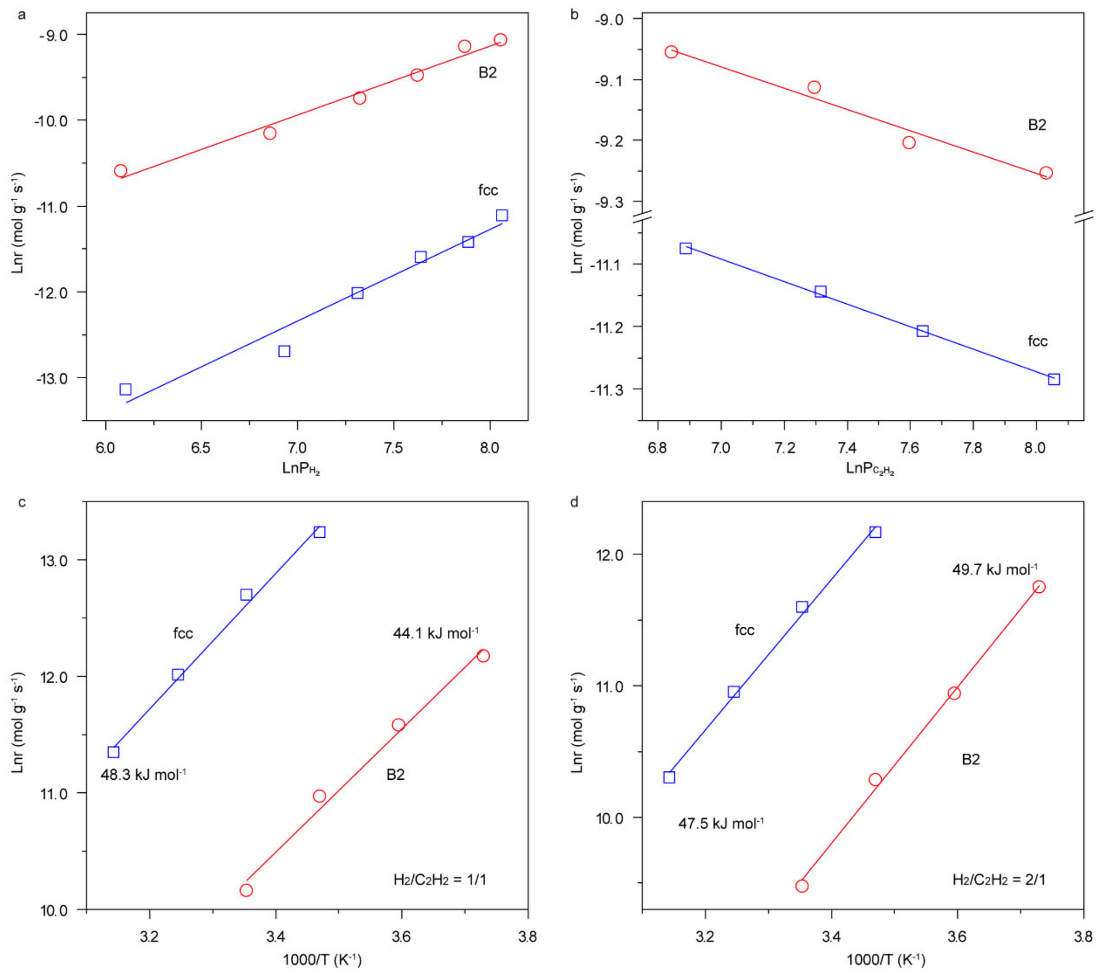

Supplementary Figure 12. Reaction kinetics over the PdCu nanoparticles. a, **b**, Reaction orders with respect to H_2 (**a**) and C_2H_2 (**b**), measured at 298 K by varying the concentrations of H_2 (0.5–3 vol.%) or C_2H_2 (1.0–3 vol.%) in the feed gases. The reaction orders are quite similar on the two particles, being 0.79-1.0 on H_2 and -0.18 on C_2H_2. The nearly first order regarding H_2 suggests that the dissociation of H_2 is the rate-limiting step while the activated H atoms are rapidly consumed by acetylene. **c**, **d**, Apparent activation energies at the H_2/C_2H_2 molar ratios of 1/1 (**c**) and 2/1 (**d**) in the temperature range 268–318 K; the fraction of C_2H_2 in the feed gas is kept at 1.0 vol.% while that of H_2 is set at 1.0 or 2.0 vol.%, balanced by He.

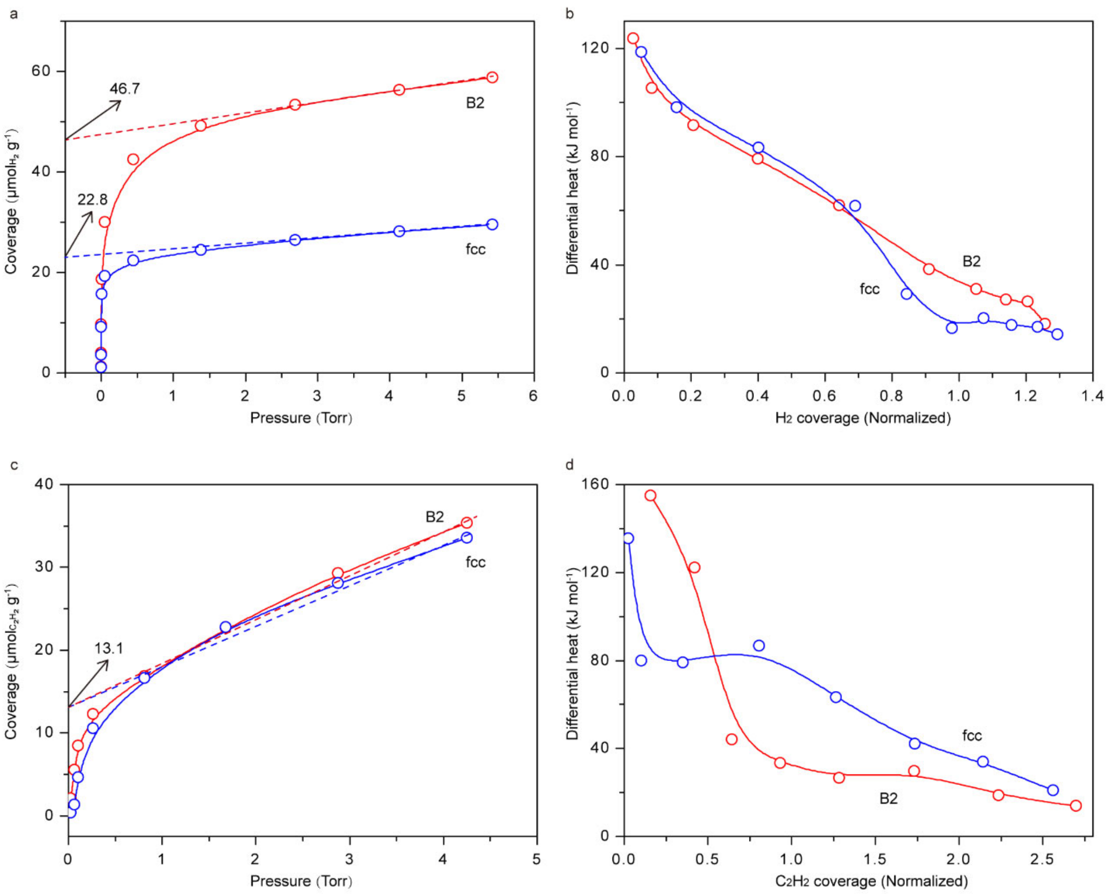

Supplementary Figure 13. Hydrogen chemisorption on the PdCu nanoparticles. a-d, Microcalorimetric adsorptions of H_2 (**a, b**) and C_2H_2 (**c, d**) on the B2 and fcc particles at 313 K. The amount of adsorbed hydrogen (**a**), at a monolayer level, on the B2 particle (46.7 μmol g^{-1}) is about twice of that over the fcc particle (22.8 μmol g^{-1}). The initial adsorption heat of H_2 (**b**) is 123 kJ mol^{-1} on the B2 particle and 118 kJ mol^{-1} over the fcc particle. Notably, the adsorption plateau, which is observed on pure Pd particles, disappears over both samples, evidencing spillover of the activated hydrogen atoms from Pd site to the particle surface. Both particles have a saturated amount of C_2H_2 adsorbed (13.1 μmol g^{-1}), suggesting a similar model of acetylene adsorption (**c**); the initial adsorption heat of C_2H_2 (**d**) on the B2 particle (154 kJ mol^{-1}) is slightly higher than that on the fcc particle (135 kJ mol^{-1}). The abrupt change in the differential heat, as increasing C_2H_2 coverage, indicates the adsorption geometry of C_2H_2 shifts from di-σ-bonded molecule to π-bonded state.

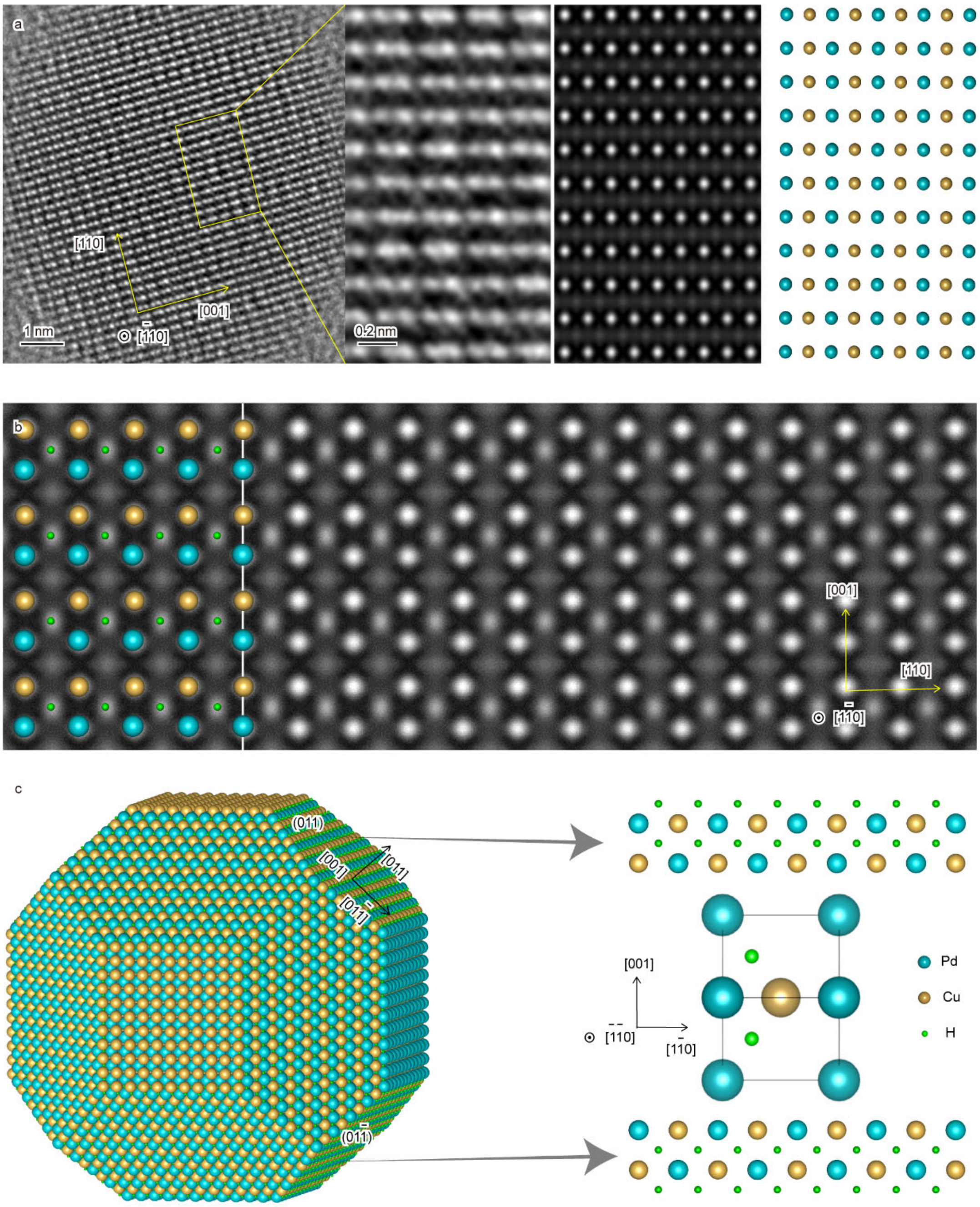

Supplementary Figure 14. Environmental TEM analysis on H_2 dissociation over the B2 particle. a, An image taken along the $[\bar{1}10]$ zone axis under vacuum at 303 K; the enlarged and simulated images of the rectangular region reaffirm the orderly-arranged Pd and Cu atomic columns (bright dots). **b**, A simulated image of the {110} facets of the B2 particle under hydrogen atmosphere, evidencing that the intensities of hydrogen atomic columns appear clearly as the number of H atoms approach 4. **c**, A schematic illustration of H_2 dissociation over a B2 particle (constructed from the STEM images in Fig. 1a-c), showing that the activated H atoms are quantified into the top/sublayers. There are 348 H atoms over each (110) facet, approximately equaling to a density of 33 H nm^{-2}.

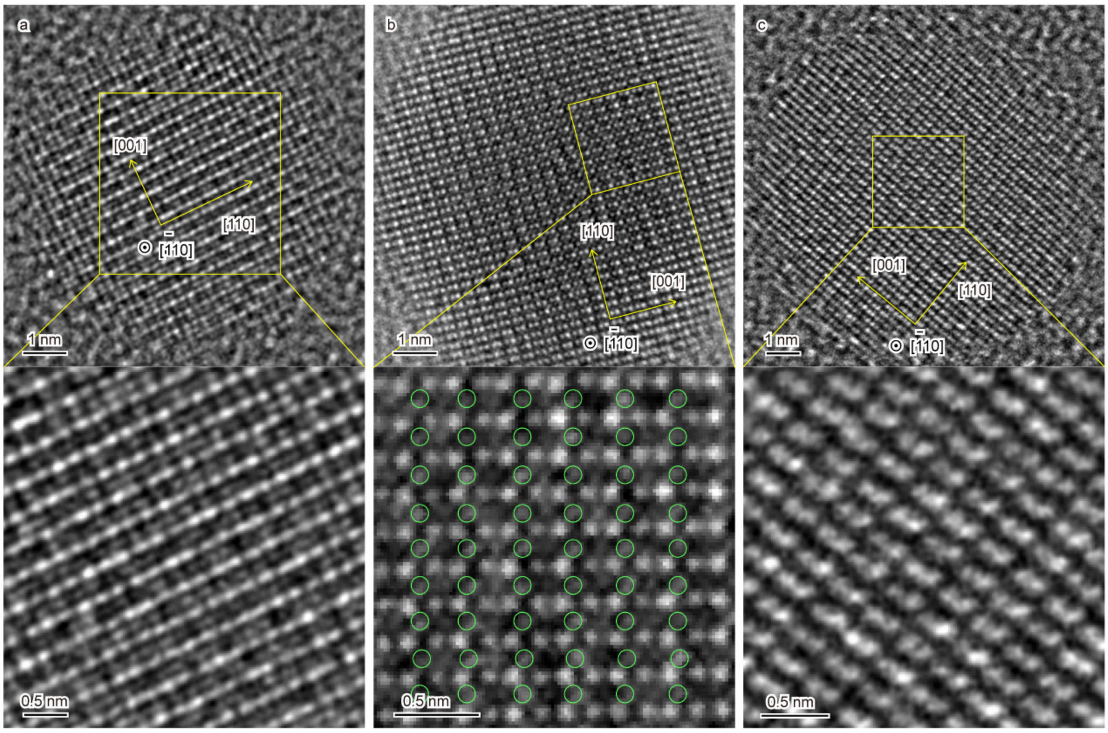

Supplementary Figure 15. Environmental TEM images of the B2 particle under H_2/C_2H_2 at 303 K. a, A TEM image taken under vacuum at 303 K; the enlarged image of the selected region shows the ordered Pd and Cu atomic columns. **b**, A TEM image acquired upon exposing the B2 particle to H_2, depicting the dissociated hydrogen atoms (green circles) that locate between Pd and Cu atomic columns. **c**, A TEM image recorded under H_2/C_2H_2 (molar ratio of 1/1), displaying the Pd and Cu atomic columns but without hydrogen atomic column. Hydrogenation of acetylene induces dynamic variations of Pd and Cu atoms. All the images are taken along the [1$\bar{1}$0] zone axis of the B2 particle.

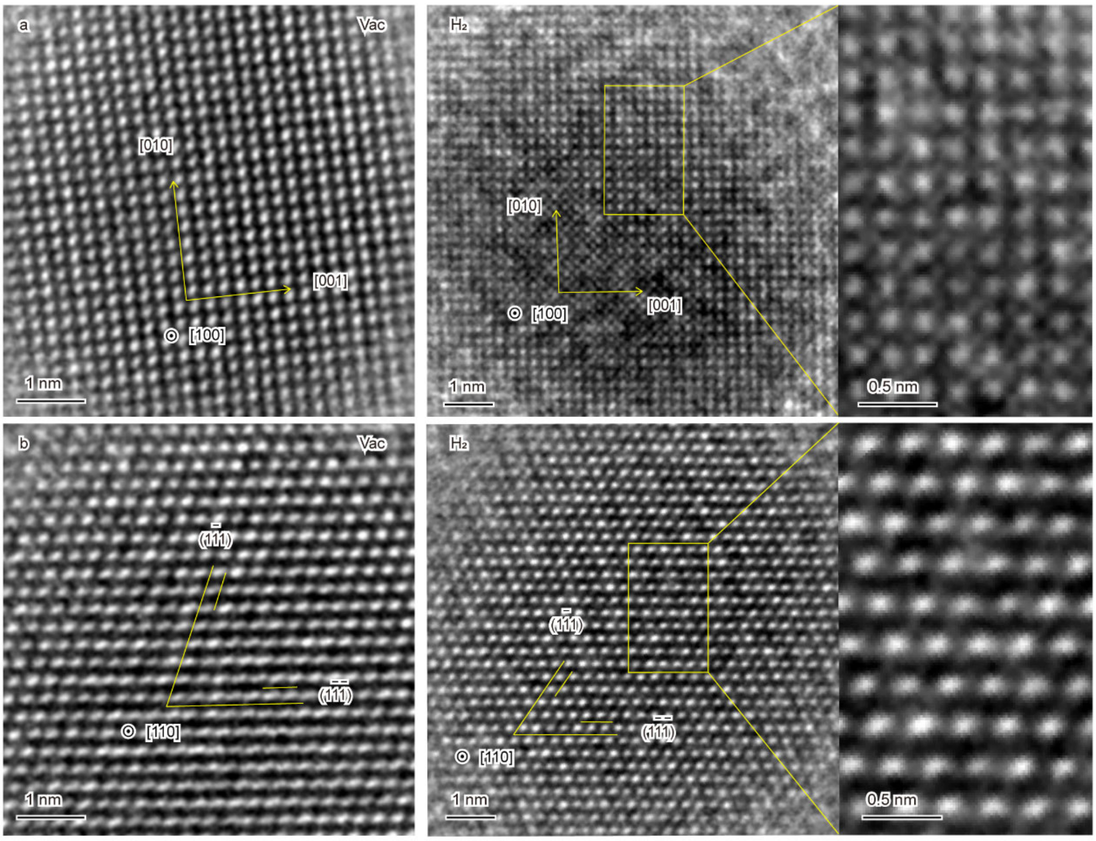

Supplementary Figure 16. Environmental TEM images of the fcc particle under H_2 at 303 K. a, **b**, TEM images taken along the [100] (**a**) and [110] (**b**) zone axis under vacuum (left) and H_2 (right); the enlarged images of the rectangular regions show the location of metal atoms on the respect facets but rarely H atoms.

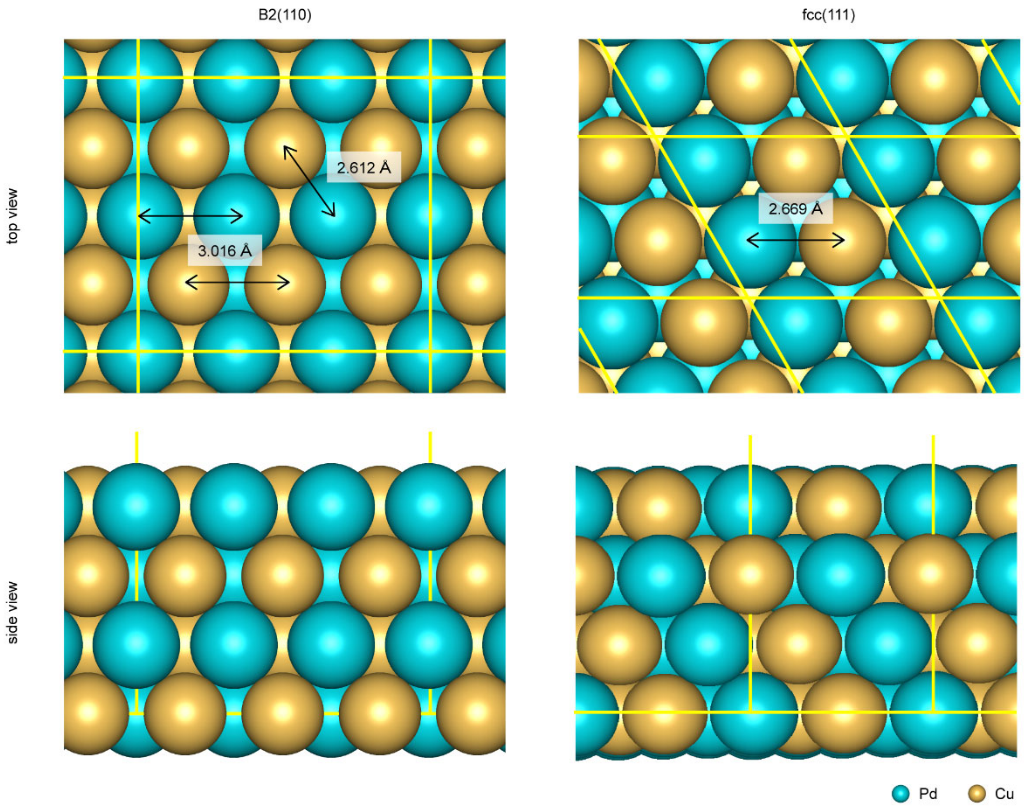

Supplementary Figure 17. Representative models of B2(110) and fcc(111) surfaces. On the surface layer of B2(110), atomic rows of Pd and Cu are arranged alternately. One surface Pd (or Cu) atom has the first nearest neighbors of 4 Cu (or Pd) atoms with Pd-Cu bond length of 2.612 Å while Pd-Pd (or Cu-Cu) coordination appears only in the second nearest neighbors with the distance of 3.016 Å. On fcc(111) surface, one Pd (or Cu) atom has the first nearest neighbors of 6 coordinations including four hetero-metallic Pd-Cu bonds and two homometallic Pd-Pd or Cu-Cu bonds at the same bond length of 2.669 Å [19,20]. The disordered fcc(111) facet was simulated by using an ordered L10-type (the AuCu-type) phase because both share the same space packing pattern of metal atoms[16,17].

Supplementary References

1 Newville, M. IFEFFIT: interactive XAFS analysis and FEFF fitting. *J. Synchrotron Rad.* **8,** 322–324 (2001).

2 Joly, Y. X-ray absorption near-edge structure calculations beyond the muffin-tin approximation. *Phys. Rev. B* **63,** 125120(1–10) (2001).

3 Wang, Y. & Wöll, C. IR spectroscopic investigations of chemical and photochemical reactions on metal oxides: bridging the materials gap. *Chem. Soc. Rev.* **46,** 1875–1932 (2017).

4 Chen, A. et al. Structure of the catalytically active copper–ceria interfacial perimeter. *Nat. Catal.* **2,** 334–341 (2019).

5 Wöll, C. Structure and chemical properties of oxide nanoparticles determined by surface-ligand IR spectroscopy. *ACS Catal.* **10,** 168–176 (2020).

6 Kresse, G. & Furthmüller, J. Efficiency of *ab-initio* total energy calculations for metals and semiconductors using a plane-wave basis set. *Comput. Mater. Sci.* **6,** 15–50 (1996).

7 Kresse, G., Joubert, D. From ultrasoft pseudopotentials to the projector augmented-wave method. *Phys. Rev. B* **59,** 1758–1775 (1999).

8 Perdew, J. P., Burke, K. & Ernzerhof, M. Generalized gradient approximation made simple. *Phys. Rev. Lett.* **77,** 3865–3868 (1996).

9 Dronskowski, R. & Blöchl, P. E. Crystal Orbital Hamilton Populations (COHP). Energy-resolved visualization of chemical bonding in solids based on density-functional calculations. *J. Phys. Chem.* **97,** 8617–8624 (1993) .

10 Deringer, V. L., Tchougreeff, A. L. & Dronskowski, R. Crystal Orbital Hamilton Population (COHP) analysis as projected from plane-wave basis sets. *J. Phys. Chem. A* **115,** 5461–5466 (2011).

11 Maintz, S., Esser, M. & Dronskowski, R. Efficient rotation of local basis functions using real spherical harmonics. *Acta Phys. Pol. B* **47,** 1165–1175 (2016).

12 Koga, T., Kanayama, K., Watanabe, T., Imai, T. & Thakkar, A. J. Analytical Hartree-Fock wave functions for the atoms Cs to Lr. *Thero. Chem. Acc.* **104,** 411–413 (2000).

13 Koga, T., Kanayama, K., Watanabe, T. & Thakkar, A. J. Analytical Hartree-Fock wave functions subject to cusp and asymptotic constraints: He to Xe, Li^+ to Cs^+, H^- to I^-. *Int. J. Quantum Chem.* **71,** 491–497 (1999).

14 Maintz, S., Deringer, V. L., Tchougréeff A. L. & Dronskowski, R. LOBSTER: A tool to extract chemical bonding from plane-wave based DFT. *J. Comput. Chem.* **37,** 1030–1035 (2016).

15 Henkelman, G. & Jónsson, H. A dimer method for finding saddle points on high dimensional potential surfaces using only first derivatives. *J. Chem. Phys.* **111,** 7010–7022 (1999).

16 Qiu, Y. et al. BCC-phased PdCu alloy as a highly active electrocatalyst for hydrogen oxidation in alkaline electrolytes. *J. Am. Chem. Soc.* **140,** 16580–16588 (2018).

17 Yuan, D., Cai, L., Xie, T., Liao, H. & Hu, W. Selective hydrogenation of acetylene on Cu-Pd intermetallic compounds and Pd atoms substituted Cu(111) surfaces. *Phys. Chem. Chem. Phys.* **23,** 8653–8660

(2021).

18 Monkhorst, H. J. & Pack, J. D. Special points for Brillouin-zone integrations. *Phys. Rev. B* **13,** 5188–5192 (1976).

19 Bozzolo, G., Garcés, J. E., Noebe, R. D., Abel, P., & Mosca, H. O. Atomistic modeling of surface and bulk properties of Cu, Pd and the Cu–Pd system. *Prog. Surf. Sci.* **73,** 79–116 (2003).

20 Yuan, D. & Zhang, Y. Theoretical investigations of HCOOH decomposition on ordered Cu–Pd alloy surfaces. *Appl. Surf. Sci.* **462,** 649–658(2018).
